# Deep Learning-Based Cattle Vocal Classification Model and Real-Time Livestock Monitoring System with Noise Filtering

**DOI:** 10.3390/ani11020357

**Published:** 2021-02-01

**Authors:** Dae-Hyun Jung, Na Yeon Kim, Sang Ho Moon, Changho Jhin, Hak-Jin Kim, Jung-Seok Yang, Hyoung Seok Kim, Taek Sung Lee, Ju Young Lee, Soo Hyun Park

**Affiliations:** 1 Smart Farm Research Center, Korea Institute of Science and Technology (KIST), Gangneung 25451, Korea; jeoguss@gmail.com (D.-H.J.); cto@1778tech.com (C.J.); inenviron@kist.kr (J.-S.Y.); hkim58@kist.re.kr (H.S.K.); tslee@kist.re.kr (T.S.L.); jyl7318@kist.re.kr (J.Y.L.); 2 Department of Bio-Convergence Science, College of Biomedical and Health Science, Konkuk University, Chungju 27478, Korea; narziss924@hanmail.net (N.Y.K.); moon0204@kku.ac.kr (S.H.M.); 3 Asia Pacific Ruminant Institute, Icheon 17385, Korea; 4 Department of Smartfarm Research, 1778 Living Tech, Sejong 30033, Korea; 5 Department of Biosystems and Biomaterial Engineering, College of Agriculture and Life Sciences, Seoul National University, Seoul 08826, Korea; kimhj69@snu.ac.kr

**Keywords:** cattle vocalization, sound classification, MFCC, convolutional neural network

## Abstract

**Simple Summary:**

In the application of artificial intelligence and advanced sound technologies in animal sound classification, certain challenges are still faced, such as the disruptions of background noise. To address this problem, we propose a web-based and real-time cattle monitoring system for evaluating cattle conditions. The system contained a convolutional neural network (CNN) for classifying cattle vocals and removing background noise as well as another CNN for behavior classification from existing datasets. The developed model was applied to cattle sound data obtained from an on-site monitoring system through sensors and achieved a final accuracy of 81.96% after the sound filtering. Finally, the model was deployed on a web platform to assist farm owners in monitoring the conditions of their livestock. We believe that our study makes a significant contribution to the literature because it is the first attempt to combine CNN and Mel-frequency cepstral coefficients (MFCCs) for real-time cattle sound detection and a corresponding behavior matching.

**Abstract:**

The priority placed on animal welfare in the meat industry is increasing the importance of understanding livestock behavior. In this study, we developed a web-based monitoring and recording system based on artificial intelligence analysis for the classification of cattle sounds. The deep learning classification model of the system is a convolutional neural network (CNN) model that takes voice information converted to Mel-frequency cepstral coefficients (MFCCs) as input. The CNN model first achieved an accuracy of 91.38% in recognizing cattle sounds. Further, short-time Fourier transform-based noise filtering was applied to remove background noise, improving the classification model accuracy to 94.18%. Categorized cattle voices were then classified into four classes, and a total of 897 classification records were acquired for the classification model development. A final accuracy of 81.96% was obtained for the model. Our proposed web-based platform that provides information obtained from a total of 12 sound sensors provides cattle vocalization monitoring in real time, enabling farm owners to determine the status of their cattle.

## 1. Introduction

Global meat consumption has increased rapidly because of a population increase and rapid economic growth. To meet the increasing demand for livestock products, the livestock industry has expanded and implemented dense breeding. Additionally, various information communications technologies have aided the development of precision livestock farming (PLF), through which the livestock industry is pursuing welfare breeding (which improves livestock rearing environments) and the production of quality livestock products [1,2]. The vital part of such PLF is the technology to accurately monitor the current conditions of the livestock.

Vocal information is an effective communication tool between animal groups or individuals and has the advantage of effectively reaching a long range. The voice provides information about the age, gender, sequence, and breeding status of the vocalizing animal. The voice of cattle, therefore, contains information about the animal’s extraordinary conditions, such as pain, estrus, separation from the calf, and hunger or thirst [3,4]. When the cattle feel hungry or thirsty, or are is in estrus [5] or a certain stress situation, the cattle phonate a voice with a specific pattern.

Many studies have been conducted to extract the features of these acoustic sounds for classification. Kiley [6] first recorded and analyzed the vocalizations of cattle and found that in a herd of mixed-breed beef and dairy cattle, there were six distinct call types comprising different combinations of five syllables. Watts and Stookey [7] argued that vocal behavior in cattle may be interpreted as a form of subjective commentary by an individual animal on its own internal condition. These findings are more thoroughly investigated and understood, and will be a valuable resource for cattle welfare monitoring [8]. Recent advances in audio and video recording technology have also facilitated active research on animal phonetics. Meen et al. [2] reported a potential welfare monitoring system that observes the vocalizations and behaviors of Holstein Friesian cattle using audio and video recordings. Röttgen et al. [9] reported that vocalization rate was a suitable indicator to confirm cattle’s estrus status, and it was suggested that the status of cattle can be monitored through voice analysis. According to Bishop et al. [10], numerous studies on PLF have proposed methods of extracting audio-specific features of animals. Moura et al. [11] proposed a sound acquisition system for distinguishing piglet vocalizations in stressful and nonstressful situations by relative sound intensity. Similarly, Fontana et al. [12] used peak frequency to classify broiler vocalizations. There have also been many attempts to analyze vocal data by converting them to a frequency domain such as a Fourier transform [13,14,15]. The Mel-frequency cepstral coefficient (MFCC) feature extraction technique includes windowing the signal, applying the discrete Fourier transform, taking the log of the magnitude, and warping the frequencies on a Mel scale. Afterward, the inverse discrete cosine transform is applied. It has been reported that audio data converted to MFCC can be effectively classified for sound classification [16], because the potentially available phonetic information is included to facilitate feature classification. Therefore, MFCCs have acoustic features that have been widely used in various applications [10,17,18,19]. As deep learning-based classification models have shown capabilities of high accuracy and reliability in various fields [20,21], the models have a high potential for effectively classifying animal vocals that contain behavioral meanings. Environmental sound recognition is an important pattern recognition problem because artificial intelligence is becoming widely adopted for protecting biodiversity and conservation [22]. Animal voices have been used to conduct studies on animal species classification [23], whereas classification studies on the behaviors and statuses of specific animals have been rarely conducted [24].

A convolutional neural network (CNN) is a deep learning technology in which a data array of two or more dimensions, such as an image, is stacked through a plurality of two-dimensional filters. CNNs show high accuracies in image classification and have been recently applied in speech classification [25,26,27]. For animal sound classification using CNNs, Xie and Zhu [28] applied deep learning in classifying Australian bird sounds and reported a classification accuracy of more than 88%. Xu et al. [29] proposed a multiple-view CNN architecture to classify species of animals using a wireless acoustic sensor network. Although advancements in this classification technology enable real-time monitoring of voice data in the field of PLF, the maintenance of quality in the vocal acquisition system remains a problem [30,31,32].

Because most livestock facilities are semiclosed systems, they are not completely isolated from outside noises. Thus, a device for acquiring and monitoring sound in a livestock house must separate the livestock noises from external noises. There is a high tendency to encounter external noises, such as white noise, in analog signal collection because of the dust generated in livestock houses, equipment aging, and a poorly equipped electrical signal shielding system. When these noises are not filtered out, they pose a major problem in voice monitoring systems. Chedad et al. [33] proposed a method for distinguishing cough sounds from other sounds, such as grunts, metal clanging, and background noise, using neural networks for the classification. Recent cases of effective noise reduction using short-time Fourier transform (STFT) filters have also been reported in several studies [34,35].

Therefore, in order to effectively acquire sound and accurately determine this information in a livestock facility, artificial intelligence technology is required to determine whether it is a cattle’s voice by acquiring noise-removed voice information and analyzing it. This present study aimed to develop a monitoring system for classifying sounds by effectively acquiring cattle’s voices in livestock facilities. Thus, we propose a deep CNN model (1) for classifying cattle cries and other sounds by removing noises from the voices by using an STFT filter and a CNN model (2) for behavioral classification using previously acquired data of the classified cattle voice model. By installing this analysis model on a web-based monitoring system, we intend to construct a system with the potential to grasp the condition of livestock through the analysis of recorded sounds generated in the livestock house and ultimately contribute to livestock welfare.

## 2. Materials and Method

### 2.1. Description of On-Site Sound Monitoring System

**Figure 1:**
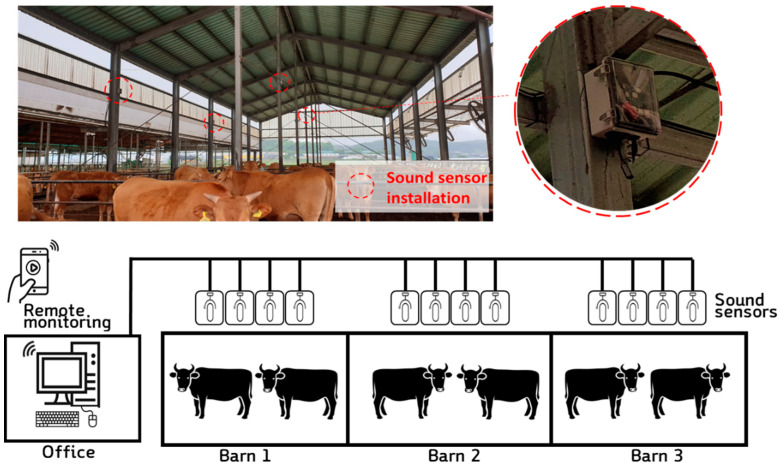
The installed vocal recording devices and web-based monitoring system.

The sound monitoring device and system were configured to effectively record and analyze sounds from individual cattle using monitoring sensors capable of recording sound through a microcontroller and a microphone. The microcontroller was used to collect the sound data and perform preprocessing to filter the voice data. As shown in Figure 1, the system was installed such that four monitoring devices were located at a height of 3 m in three separate livestock facilities, making a total of 12 monitoring devices. The apparatus for collecting the vocal data was composed of Raspberry Pi 3+, USB mic (USB mic, Shenzhen kobeton technology, Shenzhen, China, frequency response: 100 kHz to 16 kHz, sensitivity: −47 dB ± 4 dB), and a mini-PC (NUC10i5FNHJA, Intel, Santa Clara, CA, USA).

The obtained sound files contained real-time sound data, and the data were saved when the amplitude was over 60 dB (the reason for this is explained later in the noise filtering section (Section 2.2). The collected data are being saved in DB on the local server PC. If the same sound was recorded through each 4 sensors in the same zone, after checking the same sound recorded at the same time, only one sound with the highest dB is selected. Subsequently, the files, after being preprocessed through the filter, were delivered to a small PC installed in the central control office. This PC served as the database of the sound data collected from the 12 recording sensors and simultaneously ran the web-based cattle status monitoring page. Additionally, the developed deep learning model for classifying the sound class was run on the PC, which also provided information to the user through the developed web page. The monitoring page, which was based on Flask and JavaScript, uploaded the collected information in real-time and was designed to check and download the recorded files and voice information.

### 2.2. Noise Filtering Using Short-Time Fourier Transform and Mask Smoothing

An STFT filter was adopted to remove the background noise in the farm and the analog white noise of the microphone, both of which were recorded along with the cattle’s vocal sounds. The STFT filter generally improves the quality of the incoming sound signal by eliminating noises from the sound signal.

The STFT is a Fourier-related transform used to determine the sinusoidal frequency and phase content of local sections of a signal as it changes over time [36]. In practice, STFTs are computed by dividing a time signal into shorter segments of equal lengths and computing the Fourier transform separately on each shorter segment. Thus, the Fourier spectrum for each shorter segment is revealed. One then usually plots the changing spectra as a function of time; the plot is known as a spectrogram or waterfall plot.

For discrete time, the data to be converted can be divided into chunks or frames. Each chunk is Fourier transformed, and the complex result is added to a matrix that records the magnitude and phase for each point in time and frequency [37]. The discrete STFT *X* of the signal *x* is given by

(1) $$X\left(l,k\right)=\sum_{n=0}^{N-1}w\left(n\right)\times \left(n+lH\right)e^{\frac{-2\pi kn}{N}}$$

where k is the frequency axis, n is the time axis, l is the length of window, w is the window function, and H is the hop size.

**Figure 2:**
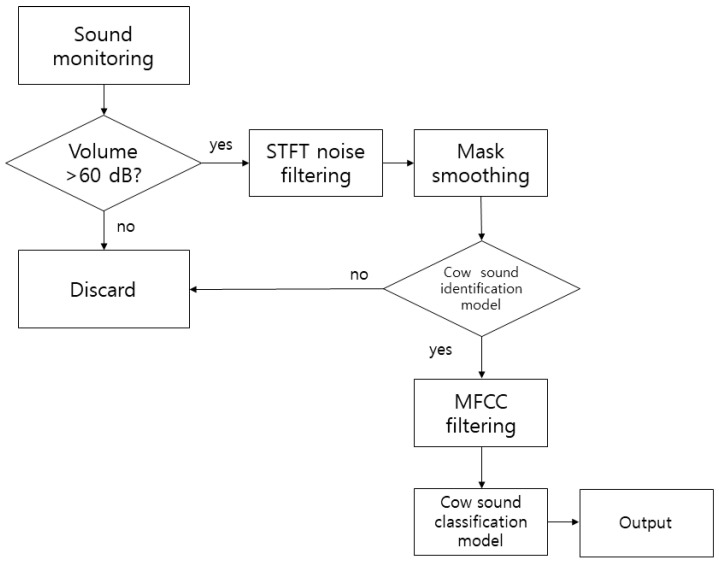
Flow chart of the audio data collection and noise filtering in the on-site monitoring system.

**Figure 3:**
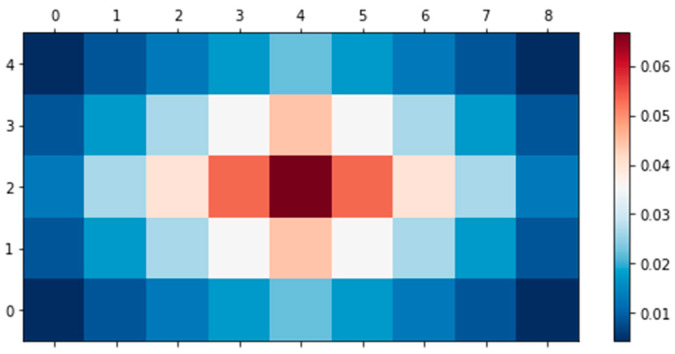
Filter for smoothing mask.

The noise removal algorithm is illustrated in Figure 2. The average hearing range of cattle for a standard 60 dB tone is between 23 and 37 kHz. The most significant sensitivity occurs at approximately 8 kHz [6,36,38]. The value of 60 dB is the reference tone proposed in this study, and when the sound sensor reacts sensitively to too little sound, excess sound data are recorded, Therefore, to separate the cattle’s voice and other noises, the recording system stores the sounds that are higher than 60 dB. When more than 60 dB of sound was generated, the audio was recorded for 5 s; the noise was then removed through STFT. The number of audio frames between the STFT columns used was 2048, the windowed length was designated as 2048, and a hop size of 256 was used. Mask audio (cattle’s voice) was processed through smoothing, excluding the noise extracted from the STFT. Here, two frequency channels and four time channels were equalized with a smoothing filter. Figure 3 is a schematic of the mask filter. We used the librosa and noisereduce libraries of Python 3.7 for this sound analysis.

### 2.3. Deep Neural Network Models for Classification

#### 2.3.1. Audio Data Conversion Using Mel-Frequency Cepstral Coefficients

To visualize the acoustic data, we preprocessed the audio data through MFCC. Mel-frequency cepstrum (MFC) is a representation of the short-term power spectrum of a sound, based on a linear cosine transform of a log power spectrum on a nonlinear frequency Mel scale. MFCCs are coefficients that collectively form an MFC [22,39]. MFCCs are derived from a type of cepstral representation of the audio clip. The difference between the cepstrum and MFC is that in the MFC, the frequency bands are equally spaced on the Mel scale, which approximates the human auditory system’s response more closely than the linearly spaced frequency bands used in the normal cepstrum. This is performed by the Mel filter bank, which is composed of triangular filters that are distributed on the Mel scale. The filter bank was calculated using Equation (2):

(2) $$B\left(m,k\right)=\{\begin{array}{c} 0,\;k<f\left(m-1\right)\\ \frac{k-f\left(m-1\right)}{f\left(m\right)-f\left(m-1\right)},\;f\left(m-1\right)\leq k\leq f\left(m\right)\\ \frac{f\left(m+1\right)-k}{f\left(m+1\right)-f\left(m\right)},\;f\left(m\right)\leq k\leq f\left(m+1\right)\\ 0,\;k<f\left(m+1\right) \end{array},$$

where B(m,k) is the matrix of filter banks, *f* is frequency, m is the number of bank filters, and k is the number of analysis windows.

A filter bank was obtained for each window of the speech signal. The first filter obtained was very narrow, indicating the amount of energy present near 0 Hz [40]. As the frequencies increase, the filters expand and the variations are smaller. To determine the energy of the filter banks, multiply each bank of filters using the power spectral density windows and add the coefficients as shown in Equations (3) and (4).

(3) $$E\left(m,k\right)=\;\sum_{m=1}^{M}B\left(m,k\right)P\left(k\right),\;k=1,2,\;\ldots ,\;k,$$

(4) $$E_{log}\left(m,k\right)=log\left(\sum_{m=1}^{M}B\left(m,k\right)P\left(k\right)\right)$$

where *P(k)* is the power spectral density and k represents the number of windows that go from the first to the k-th window of the cattle’s call. Subsequently, the logarithm of the filter bank energy was calculated [40]. This work has the effect of closely matching the obtained traits to what humans actually hear. The discrete cosine transform (DCT) of the filter bank energy log was then calculated to obtain the MFCC. The DCT was used to reduce the computational cost and is defined as shown in Equation (5).

(5) $$MFCC\left(n\right)=\sum_{m=1}^{M}E_{log}\left(m,k\right)\cos\left[n\left(m-\frac{1}{2}\right)\frac{\pi }{M}\right]$$

**Figure 4:**
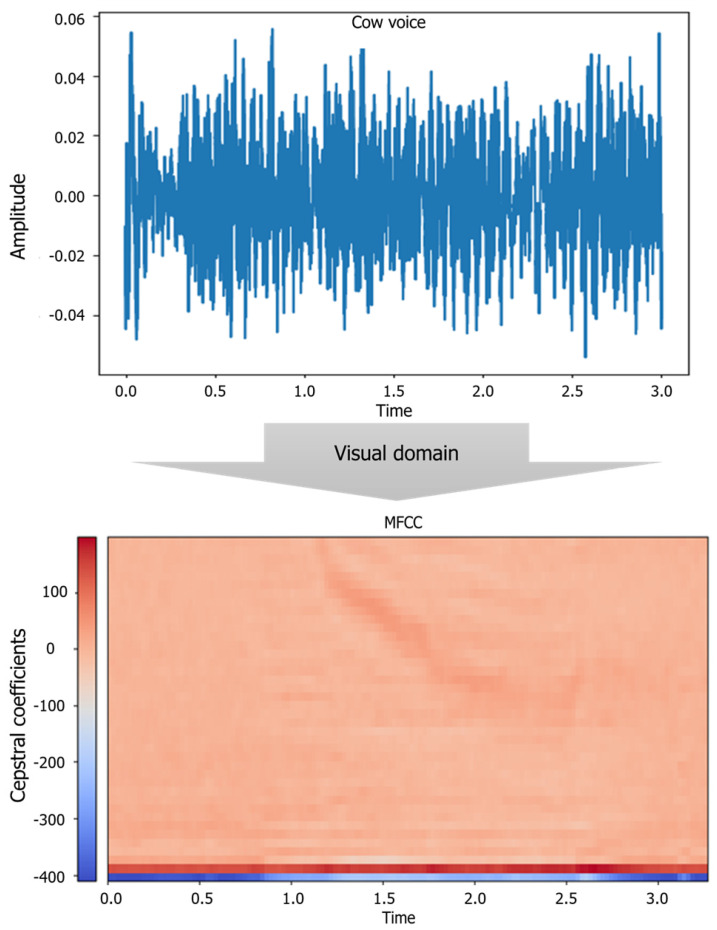
The conversion of sound information into cepstrum form using Mel-scale filter.

This frequency warping can allow for better representation of sound—for example, in audio compression [16]. Figure 4 illustrates the process of converting the cattle vocalization into MFCC in this study. In this case, the number of filter bands used was 128, the time frame was fixed to 2000, and if the sound sample was less than 2000, all sound data were assigned a value of 0. Most of the samples already recorded are between 5 and 10 s, and the 2000 frame was considered to be sufficient to be used as a constant input value to the CNN as a time equivalent to 20 s.

#### 2.3.2. CNN Model for Removing External Sounds

**Figure 5:**
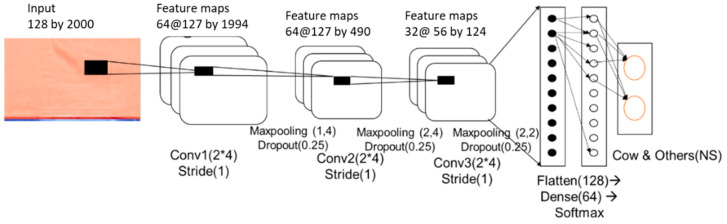
Two-dimensional convolutional neural network (CNN) model for classifying cattle voice and other noises.

A deep learning classification model was developed for classifying cattle voices and other noise samples generated in the barn environment. The sounds of the livestock environment generated at this time include noises of the microphone itself, the operation sound of the livestock farm machinery, metal clanging, bird chirping, dialog between farm workers, barking of a dog, and various other sounds. For the recorded samples, a total of 12,000 recorded files were collected from 10–20 December 2019. A CNN-based deep learning model was developed to classify the data into two classes by extracting only 677 cattle voice samples and 1000 samples for other sounds. The structure of the CNN model is depicted in Figure 5.

The classification accuracy of the developed model was evaluated using the original data of the files whose audio noises were removed and mask smoothed through the STFT filter. Precision, recall, and accuracy were used to determine the effectiveness of the two self-diagnostic methods. When evaluating the model, the relationship between the model’s predicted label and the actual correct label was considered. The result is presented as true and false as the classification model also returns true and false, dividing the case into the 2 × 2 matrix shown in Table 1.

Although accuracy is commonly used to evaluate categorization techniques, the measure adopted in this study is considerably less sensitive to variations in the number of correct decisions than to precision and recall. The accuracy ($\alpha_{i}$) is given by Equation (6):

(6) $$\alpha_{i}=\frac{TP_{i}+TN_{i}}{TP_{i}+TN_{i}+FP_{i}+FN_{i}}$$

#### 2.3.3. CNN Model for Cattle Behavioral Voice Classification

The CNN model we adopted for cattle behavioral voice classification was composed of convolution, pooling, and fully connected layers and consisted of two activation functions: a rectified linear activation unit (Relu) and Softmax. A CNN applies the same equation as an Artificial neural network’s perceptron, and it updates the parameters by training the model with weights as expressed in Equation (7) [41]:

(7) $$\theta_{t+1}=\theta_{t}+\Delta \theta_{t}$$

The Adadelta function was used to optimize the CNN model used in the actual model training, which can be seen more specifically in Equations (8)–(10) than in Equation (7). Adadelta optimization was used, and it is widely used in the field of deep learning among gradient descent-based optimization methods [42]. Adadelta is an extension of Adagrad with the aim of minimizing the intense and monotonically declining learning rate [43,44], and it limits the window of the accumulated past gradients to a fixed size rather than accumulating all past squared gradients [43]. The vector of a diagonal matrix ($E{\left[\Delta \theta^{2}\right]}_{t})$ with the decaying average over past squared gradients was represented as shown in Equation (8):

(8) $$E{\left[\Delta \theta^{2}\right]}_{t}=\gamma E{\left[\Delta \theta^{2}\right]}_{t-1}+\left(1+\gamma \right)\Delta \theta_{t}^{2}$$

The root mean squared error of the parameter updates is given as

(9) $$\mathrm{RMS}{\left[\Delta \theta \right]}_{t}=\sqrt{E{\left[\Delta \theta^{2}\right]}_{t}+\epsilon }$$

Finally, the Adadelta update rule is given by Equation (10):

(10) $$\Delta \theta_{t}=-\frac{\mathrm{RMS}{\left[\Delta \theta \right]}_{t-1}}{\mathrm{RMS}{\left[g\right]}_{t}}g_{t}$$

where θ is the weight, t is the updating step, $g_{t}$ is the gradient at time step t, ϵ is the smoothing term for avoiding a division by zero (10^−7^ was used), and γ is the momentum term (0.95 was used).

The vocal sounds of cattle were collected from the livestock house between January and December 2018. The cattle used in the experimental were Korean native cattle (*Bos taurus coreanae*): 130 breeding cattle (12–40 months old), 30 calves (6–8 months) old, 50 calves (under 6 months old), and 15 calves as dairy cattle. The breeding cattle density was maintained at 10 animals/10 m^2^, and a total mixed ration feed was fed to the cattle twice a day based on the Korean cattle specification standards.

The collected voice data were labeled into four classes by collecting the three groups of cattle’s behaviors and one normal voice of cattle. To classify the recorded sound, the voice data were used to identify the event time by analyzing the video and farm owner’s daily records; the data were also used to label the sound generated at that time. The labeled classes were investigated by animal physiological analysis through previous experiments. Supplementary Materials File 1 includes a few audio samples for four classes of sounds to confirm the quality of the acquired sounds.

- Estrus call: the sound of cattle estrus call was collected from 130 Korean cattle breeding cattle (aged 12 to 40 months). This corresponds to the sound produced by individual cows, identified as estrous cows, which are cows that have succeeded in conceiving through artificial insemination. At this time, the vocals produced by the cow through the voice were recorded each voice datum was collected. The proportion of primiparous cattle vocalizing individuals tended to be higher than that of multiparous cattle.
- Food anticipating call: the cattle were in a situation where the feeding time was delayed by up to 3 h (by more than 1 h for 30 Korean calves aged 6 to 8 months). At this time, the acquired sounds were collected and labeled as “Food anticipating call”.
- Cough sound: A recording device was installed in an area with the coughing cows and the recorded files were analyzed. The cough voices were collected under expert judgment at the point of the cow coughing.
- Normal call: The calls were not classified in these three cases and were classified into one class and labeled as “normal call.”

The classified classes and quantity for each sample are described in Table 2. Three main sounds were used to classify the meaning of the cattle’s voices. The repeated voices of the cattle under special circumstances were recorded, class labels were set, and data were collected. The other class was not classified as behavioral, but the sounds recorded from the cattle’s voices were collected and regarded as one class. The three classification classes were alerting sounds, and the farmer considered it as a class that needed management when there was such an alarm.

**Figure 6:**
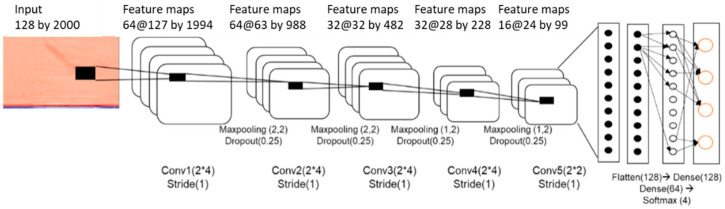
Two-dimensional CNN model for classification of cattle voice.

The behavioral voice classification model of cattle employed a CNN model with the structure shown in Figure 6. Compared with the noise classification model, the 2D convolutional layer was deeper, and the number of parameters was larger. In this model, input data identified as cattle voice were used as a model for the cattle behavioral classification. Among the 897 samples collected, 717 were used for training, and 179 samples were used for test validation.

## 3. Results

### 3.1. Noise Filter and Mask Smoothing Results

**Figure 7:**
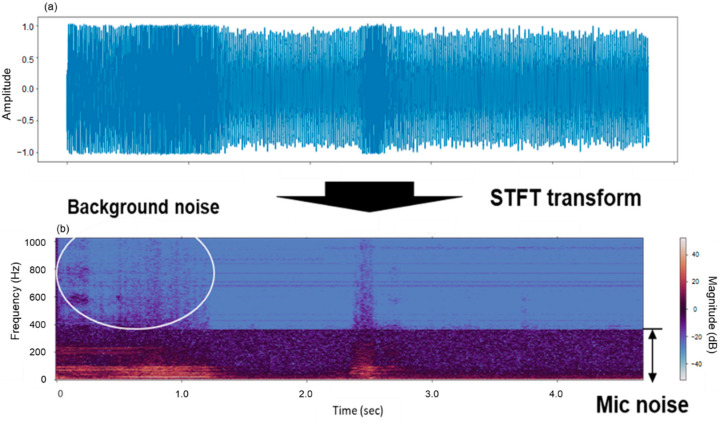
Two-dimensional source of cattle estrus call voice data (**a**) and the conversion to short-time Fourier transform (STFT) area (**b**).

In this study, sound files stored from 12 installed voice acquisition sensors were treated using STFT-based noise reduction. The effect of removing noises when applying the filter is illustrated in Figure 7, which depicts the audio sound of the cattle estrus call.

**Figure 8:**
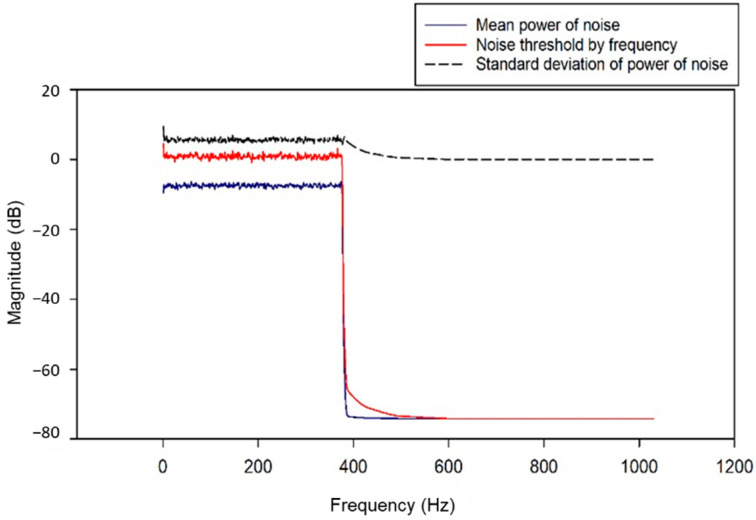
Analysis of mean-_ power and standard deviation of noise and threshold point.

**Figure 9:**
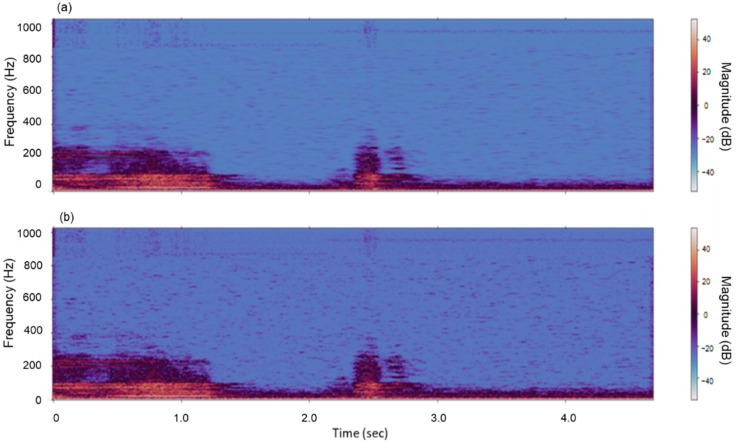
The original noise-removed audio (**a**) and voice information corrected by noise mask smoothing (**b**).

The bottom part of Figure 7 depicts the sound and voice information in the STFT area, and it can be seen that the microphone’s white noise is continuously detected below approximately 400 Hz. Additionally, the background noise around the farm was detected in the first half of the voice, but the amplitude was weak. Cattle’s voice was observed in the range of approximately 2 to 3 s. The result of measuring the parameters of noise components through STFT analysis is shown in Figure 8. The noise thresholds were obtained through mean power and standard deviation corresponding to noise components for each frequency band which has a different range for each audio sample. The noise-removed voice is omitted as shown in Figure 9a; Figure 9b depicts the STFT analysis after applying the correction effect to the noise-removed voice information through mask smoothing. This process was applied to all speech samples, and a performance comparison of the deep learning classification model was performed before and after the noise filtering. The first model is a classification model of cattle voices and farm noises.

### 3.2. Deep Neural Network Classification Performance

**Figure 10:**
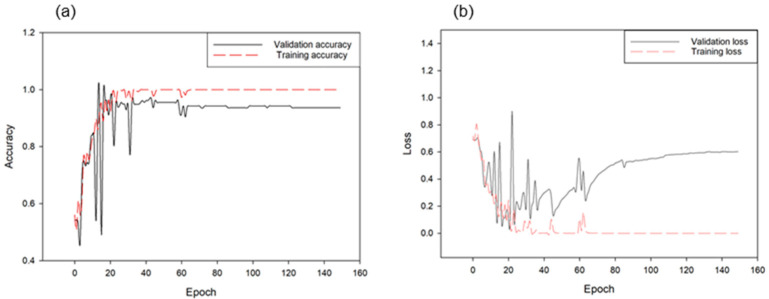
Accuracies of training and validation for the CNN model for classifying cattle voices and other sounds (**a**) as well as corresponding loss changes (**b**) (1.0 is 100% accuracy in this graph).

**Figure 11:**
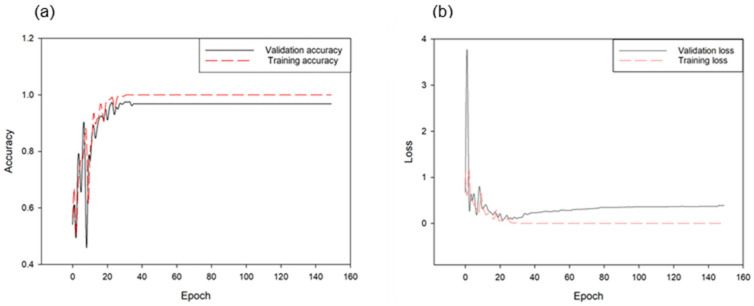
Accuracies of training and validation of the CNN model for classifying noise-filtered speech and other sounds (**a**) as well as the corresponding loss changes (**b**) (1.0 is 100% accuracy in this graph).

From the first model, approximately 360 samples were prepared as a validation set from 1667 cattle sounds and other sound classifications, and the classification accuracy of the trained model was compared in Figure 10 and Figure 11. The CNN model achieved more than 99.9% accuracy in the training and 91.38% in the validation. Table 3 presents the classification results of the CNN model in cattle vocalization in terms of true negative, false positive, false negative, true positive, false recognition rate, and true recognition rates. For both models, the true recognition rate was slightly higher than the false recognition rate. For example, in the classification between cattle voice and other sounds, the true recognition rate was 92.10%, the false recognition rate was 90.86%, and the total accuracy was 91.38%. The measurement error between the two classified samples was negligible.

By training the same CNN model through noise filtering using the same sample, slightly improved classification results were obtained. As shown in Figure 11, a 99.9% accuracy was obtained for the training set and approximately 94.18% accuracy for the validation set.

**Figure 12:**
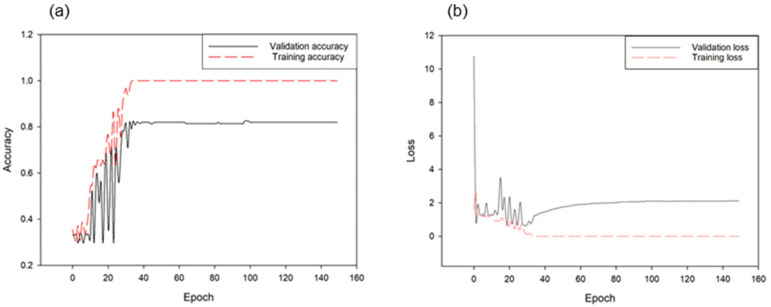
Accuracies of training and validation through the CNN model for behavioral classification of four cattle voices (**a**) and corresponding loss changes (**b**) (1.0 in this graph is 100% accuracy).

**Figure 13:**
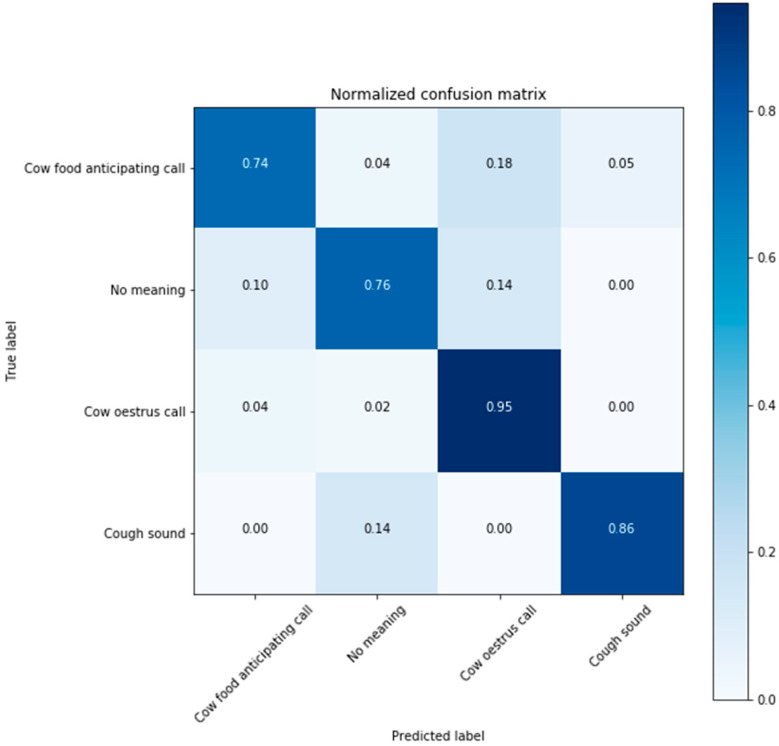
Confusion matrix of the CNN model classification accuracy for four behavioral cattle voices.

Figure 12 illustrates the results of learning the voice information of four behavioral classifications of cattle through the CNN model. The classification accuracy of the four classes was approximately 81.96%. For this model, the accuracy converged after approximately 40 epochs to complete the fitting model. Figure 13 depicts the classification accuracy of each of the four classes as on the verification set. As shown in the graph, the accuracy of the voice produced by the cattle at estrus was highest at 95.4%; similarly, it was approximately 86% for the coughing sound, 74% for the cattle’s feed-based sound, and 76% for sounds without special meaning. Particularly, approximately 18% and 14% of the cases were situations in which the feed reclining sound and normal cattle’s voices were mistaken for estrus sound, respectively.

### 3.3. Developed Web-Based Sound Information Monitoring System

**Figure 14:**
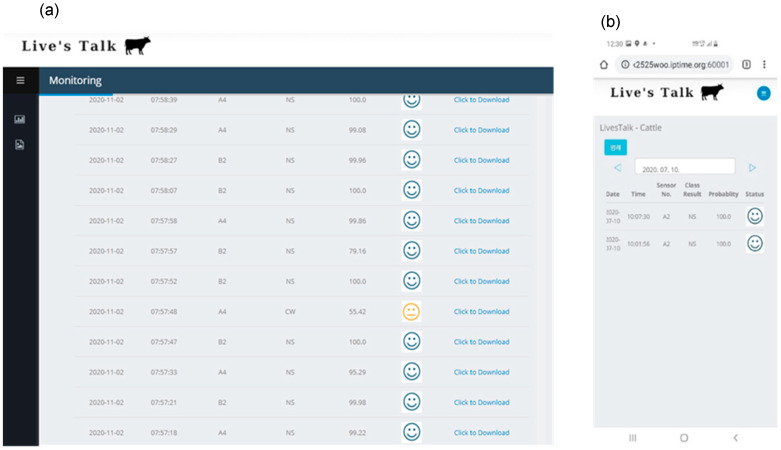
Sound monitoring analysis: WEB UI appearance (**a**) and mobile view (**b**).

The developed noise filtering and cattle voice prediction model was provided on a web platform, as shown in Figure 14. The web phase was configured to enable the farm owner see the time of the collected sound voice, the class of the sound voice predicted through the CNN model, and the probability of the corresponding class. The web platform was configured to be mobile-friendly.

## 4. Discussion

For speech classification using deep learning, studies have shown that RNN and CNN models achieve excellent performance in the case of Google’s VGGnet [45,46]. However, it is difficult to directly compare VGGnet with our work because the sound classification sample classes of Google were diverse. Nevertheless, the implication is that the classification accuracy of the audio class improved compared with the existing deep learning methods. The sounds of various bird species have been classified, which is a case of intensive animal sounds classification [47]. Nanni et al. [45] compared the performances of deep learning technology on the representative public animal sound datasets; the performance comparison is summarized in Table 4. This table is a representative reference for the animal speech classification accuracy of the existing deep learning technologies. Additionally, exhaustive tests have been performed on the fusion between an ensemble of handcrafted descriptors and an ensemble system based on a CNN [48], and the possibility was higher for the classification performance of the CNN-based ensemble system.

Recent attempts have been made to analyze livestock voices in relation to animal welfare in livestock facilities. A case study has also been conducted to determine poultry eating behavior from vocalization signals using deep learning [52]. Similarly, more studies are examining the correlation between the voice of a specific animal and certain behavioral meanings. This present study is a case of applying a CNN model for classification through MFCC, which is an approach that has not been previously attempted for the behavioral classification of cattle. It was significant in the collection of animal ecological speech samples, and statistical significance could not be determined by analyzing the characteristics of the existing sound parameters (maximum frequency, maximum frequency band, average amplitude, etc.).

Because various sounds are detected in the barn’s open area, it is difficult to extract the voices of cattle. To solve this problem, noise filtering technology was applied, and a model for classifying cattle vocalization and other sounds was first applied. A second model was used for the behavioral classification of the cattle’s voices. Notably, the four behavioral classification accuracies of cattle voices were approximately 81.96%. One of the classified sounds was normal sound, and the correct recognition rate was approximately 76%. This sound was installed and obtained in situations where the behavioral meaning could not be determined from the information obtained through the sensors. However, the sound may overlap with those of another class, and the most likely sound is the horn sound. Although it is outside the scope of this study, there is the possibility of distinguishing between individuals through deep learning models in the phonetic classification of cattle. In this study, the difference between individual cows was excluded from the model training. Hence, analyzing these parts with the video image is expected to yield better results in future research.

## 5. Conclusions

In this study, a deep learning speech classification model was developed to determine the status of cattle by monitoring the voices of cattle in a livestock facility. Classification of cattle noises and other noise as well as behavioral classification between cattle sounds were performed; the accuracy of each was 91.38%. The noise in the sound was removed through STFT analysis, after which the performance improved to approximately 94.18% in the classification of cow sounds and other sounds. The voices detected as cattle’s vocals were used to monitor the current cow status through a four-behavioral-classification model, and the final classification accuracy of the developed model was 81.96%.

Finally, the developed model was deployed as a web platform to provide useful information to farm owners by classifying voice files obtained from 12 sound measuring sensors installed in a livestock facility and visualizing the information on the web. It is expected that these attempts will contribute to the welfare of animals in the future.

## Figures and Tables

**Table 1:** Confusion matrix for the diagnostic model evaluation.

Model Classification Results	Actual Results	
	True	False
True	True positive (TP)	False positive (FP)
False	False negative (FN)	True negative (TN)

**Table 2:** Categories of animal sounds (*n* classes for cattle).

Index	Value	Sample Quantity
0	Estrus call	207
1	Cattle food anticipating call	178
2	Cough sound	56
3	Normal call	456

**Table 3:** Accuracy of the CNN model for cattle vocalization classification without noise filtering.

	Without Noise Filtering	With Noise Filtering
True positive	140	141
False positive	12	11
True negative	189	199
False negative	19	10
True recognition rate (%)	92.10	92.76
False recognition rate (%)	90.86	95.21
Accuracy, $\alpha_{i}$ (%)	91.38	94.18

**Table 4:** Summary of prediction accuracies of animal sound classification.

Animals or Dataset	Classification Target	Approach	Descriptor	Accuracy (%)
BIRD [49]	Forty-six species	Handcrafted features with SVM	BSIF	88.8
WHALE [48]	Whale identification	Deep learning	CNN	97.8
BIRDZ [50]	Eleven bird species		Vgg-19	96.6
Cow [19]	Oestrus detection	Ensembles of deep learning	Fus_Spec + Fus_Scatter + CNN	98.7
Sheep, cattle, dogs [30]	Classification between three animals’ vocal	MFCC with SVM	Correlation-based Feature Selection	Over 94 accuracy
Chicken [51]	Avian-influenza detection	MFCC with SVM	Discrete wavelet transform	At least 95.78 (cattle)
Chicken [52]	Eating behavior	Deep learning	PV-net	96.0

## Data Availability

The data presented in this study are available on request from the corresponding author.

## Supplementary Materials

The following are available online at https://www.mdpi.com/2076-2615/11/2/357/s1, Supplementary File 1: A few sound samples used for checking the audio quality.
